# Key community eye health messages

**Published:** 2019-05-13

## Myopia is an epidemic that needs to be managed

**Figure:**
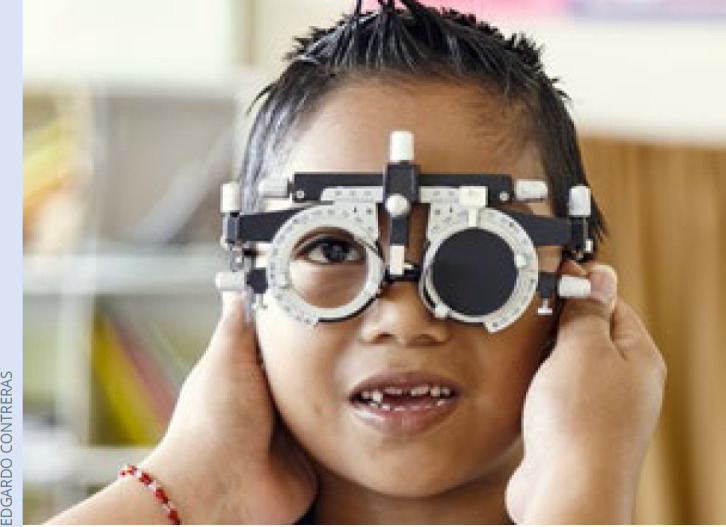

- Uncorrected myopia is the leading cause of avoidable blindness worldwide.
- Myopia (≤ −0.50 D) and high myopia (≤ −5 D) is on the increase. By 2050, half the global population could have myopia.
- This will place a huge burden on already overstretched health budgets, not only to provide spectacle correction, but also to treat the potentially blinding conditions caused by high myopia.

## The onset of myopia can be prevented or delayed

**Figure:**
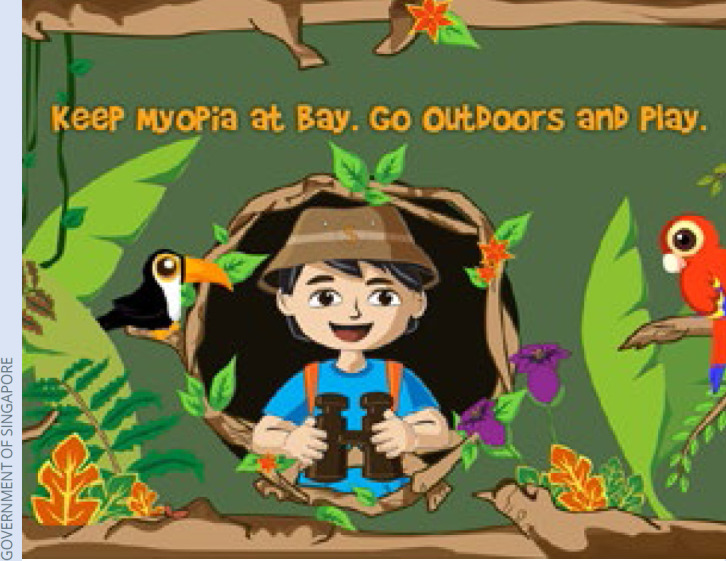

- Spending more time outdoors and less time doing close work can prevent or delay the development (onset) of myopia.
- Myopia progresses faster in younger children. Progression slows down during adolescence and ends in early adulthood.
- As a result of faster progression and more time during which progression can take place, younger children are more likely to eventually develop high myopia.

## Myopia can be managed and progression slowed down

**Figure:**
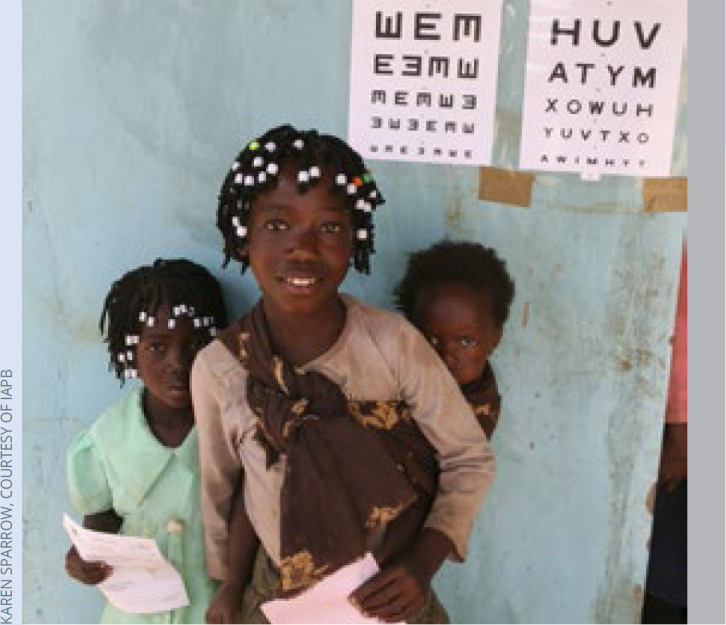

- Children with myopia need spectacles in order to achieve at school. Early detection and referral is essential, and school eye heath programmes play an important role.
- Slowing down progression reduces the risk of developing high myopia. Interventions incude daily low-dose atropine, bifocals and orthokeratology lenses. Time outdoors does not slow down progression.
- It is important to measure myopia progression in order to check how effective a particular intervention is.

